# Identification of Anthocyanin Compounds in Butterfly Pea Flowers (*Clitoria ternatea* L.) by Ultra Performance Liquid Chromatography/Ultraviolet Coupled to Mass Spectrometry

**DOI:** 10.3390/molecules26154539

**Published:** 2021-07-27

**Authors:** Nguyen Minh Thuy, Vo Quang Minh, Tran Chi Ben, My Tuyen Thi Nguyen, Ho Thi Ngan Ha, Ngo Van Tai

**Affiliations:** 1 Department of Food Technology, College of Agriculture, Can Tho University, Can Tho City 900000, Vietnam; benb1700192@student.ctu.edu.vn (T.C.B.); ntmtuyen@ctu.edu.vn (M.T.T.N.); vantai@ctu.edu.vn (N.V.T.); 2 Department of Land Resources, College of Environment and Natural Resources, Can Tho University, Can Tho City 900000, Vietnam; vqminh@ctu.edu.vn; 3 Department of Food and Nutrition, Chungnam National University, Daejeon 34134, Korea; 4 Faculty of Agriculture and Natural Resources, An Giang University, Long Xuyen City 90100, Vietnam; htnha@agu.edu.vn; 5 Vietnam National University Ho Chi Minh City, Ho Chi Minh City 700000, Vietnam

**Keywords:** anthocyanin, butterfly pea flowers, cyanidin, delphinidin, UPLC/UV/MS

## Abstract

Butterfly pea flower have great sensory attraction, but they have not yet been used widely in Vietnam. Extracts of butterfly pea flowers can be used conveniently as a natural blue colorant for food products. In this study, the identification of anthocyanin compounds in butterfly pea flowers was performed by UPLC coupled with a UV and Mass spectrometer instrument. Positive and negative ion electrospray MS/MS chromatograms and spectra of the anthocyanin compounds were determined. By analyzing the chromatograms and spectra for each ion, five anthocyanins were identified in the butterfly pea flower extract; these were delphinidin-3-(6″-*p*-coumaroyl)-rutinoside, cyanidin 3-(6″-*p*-coumaroyl)-rutinoside, delphinidin-3-(*p*-coumaroyl) glucose in both *cis*- and *trans*- isomers, cyanidin-3-(*p*-coumaroyl-glucoside) and delphinidin-3-pyranoside. Additionally, based on their intensity, it was determined that cyanidin-3-(*p*-coumaroyl-glucoside) was the most abundant anthocyanin, followed by cyanidin 3-(6″-*p*-coumaroyl)-rutinoside, delphinidin-3-(*p*-coumaroyl-glucoside), delphinidin-3-(6″-*p*-coumaroyl)-rutinoside and delphinidin-3-pyranoside. In this study, cyanidin derivatives were discovered in butterfly pea flower extract, where these compounds had not been detected in previous studies.

## 1. Introduction

Butterfly pea flowers, known *Clitoria ternatea* L., area plant species belonging to the Fabaceae family [1]. In Vietnam, butterfly pea flowers are usually used as food drinks and as a colorant. Delphinidin is the main anthocyanin responsible for the deep blue to purple color in this flower [2]. The different flower colors are mainly due to the chemical structure of the different anthocyanins or anthocyanidins synthesized in the flower [3]. In the discussion of copigmentation reactions and color stability of berry anthocyanins, it was observed that the color of anthocyanins changes from pink to blue as the number of hydroxyls increases and methoxyl groups replacing the hydroxyls reverse the trend [4]. There is increasing interest in the search and use of natural colorants, of which blue is rare and tends to be sensitive to processing and storage conditions [5]. Butterfly pea flower extract can be used as a natural blue colorant, is convenient to use, and has a longer shelf-life than equivalent plant-based colorants [6]. Therefore, it is very important to identify the main coloring compounds in butterfly pea flowers.

Anthocyanins present in edible fruits, vegetables and flowers have protective effects against diseases, especially cardiovascular disease, certain types of cancer [7], and against several chronic diseases, such as hyperglycemia [8]. Anthocyanins also improve vision [9]. Because of their benefits, anthocyanins are becoming increasingly commercializedand used in foods [10]. Anthocyanins are an important class of water-soluble pigments belonging to the flavonoid family [11]. To date, more than 600 different anthocyanins have been characterized [12]. Structurally, anthocyanins are glycosylated polyhydroxy and polyethoxy derivatives of flavylium salts; they also possess an aglycone form called anthocyanidins. Cyanidin, delphinidin, pelargonidin, peonidin, malvidin, and petunidin are the most common anthocyanidins distributed in the plants [13]. Their different structures depend on the number and position of the hydroxyl and methoxyl groups on the flavilium ring. Normally, anthocyanidins are bound to sugars, giving stability and water solubility to the molecule [14]. Among the sugars, the most common are glucose, galactose, arabinose, rhamnose and xylose, which are usually present as 3-monoglycosides and 3,5-diglycosides. Other sugars such as rutinoside (6-*o*-α-l-ramnosyl-d-glucoside), sophorosides (2-OD-glucosyl-d-glucosides) and sambubiosides (2-*o*-β-d-xylosyl-d-glucosides) [15,16] are also present.

Anthocyanins are much more soluble and stable in water than anthocyanidins. Therefore, glycosylated forms of anthocyanidins are more common in nature, and aglycones are virtually non-existent in vivo. It has been observed that glycosyl substitution stabilizes anthocyanin molecules [4]. The glycosyl units and acyl groups attached to the aglycone and their binding sites have a significant influence on the stability and reactivity of the anthocyanin molecule [17]. In addition, the number and position of the hydroxyl and methoxyl groups in the aglycone influence the chemical behavior of the pigment molecule. Increased hydroxylation of aglycone stabilizes anthocyanidins, delphinidin is more stable than cyanidin in acidic methanol. However, increasing the methylation of hydroxyl groups will weaken the stability of anthocyanins. Several anthocyanins have been identified with special structures in butterfly pea flowers. The structure of ternatin D1 was identified as delphinidin-3-*o*-(6-*o*-malonyl-β-d-glucopyranosyl)-3′, 5′-di-*o*-(-6-*o*-(E-4-)-*o*-(6-OE*p*-coumaroyl-β-d-glucopyranosy)-*p*-coumaroyl)-β-d-glucopyranoside [18]. The structure of deacylternatin was identified as delphinidin-3,3′, 5′-tri-*o*-β-d-glucopyranoside [19]. Terahara et al. [20] also determined the structure of five ternatins (A3, B4, B3, B2 and D2) as delphinidin 3-malonylG with 3′-GCG-5′-GCG, 3′-GCG-5′- GC side chains. 3′-GCGCG-5′-GC, 3′-GCGC-5′-GCG and 3′-GCGC-5′-GC, where G is d-glucose and C is *p*-coumaric acid.

Eight anthocyanins 1 to 8 (ternatins C1, C2, C3, C4, C5, D3 and preternatins A3 and C4) were isolated by Tehara et al. [21]. Structures 1–6 are recognized as delphinidin 3-malonylglucoside with 3′-GCGC-5′-G, 3′-GCGCG5′-G, 3′-GC-5′-G, 3′-GCG-5′-G, 3′-G-5′-G, and 3′-GC-5′-GC, and compounds 7 and 8 as delphinidin 3-glucoside with 3′-GCG-5′-GCG and 3′-GCG-5′-G are the side chains, respectively. Then, Nair et al. [1] went on to identify other delphinidin derivatives. Azima et al. [6] also found only anthocyanidin, delphinidin, but not cyanidin, as in some other ingredients containing anthocyanins. In addition, more recently, Esher et al. [22] identified a simple derivative of delphinidin, delphinidin-3-*o*-glucoside. The difference between the results obtained is due to different strains of *Clitoria ternatea* with different petal colors and sensitivities of analytical methods. This has been demonstrated by the study of Kazuma et al. [3], where this author identified different anthocyanins in different lines of pea flowers and they are also complex derivatives of delphinidin.

Despite many studies regarding anthocyanins in berries [23,24], studies on anthocyanins in butterfly pea flowers are still limited. In this study, anthocyanin composition in butterfly pea flowers (grown in Can Tho, Vietnam) was determined by UPLC/UV/MS for the first time.

More recently, faster methods have been proposed for the quantification of polyphenols using more powerful chromatographic systems such as UHPLC in combination with a UV detector [25,26] or mass analyzer [27]. The aim of this study was to develop a UPLC method using a UV detector and an ESI-triple quadrupole (QQQ) mass analyzer for the simultaneous determination of the major anthocyanin compounds of butterfly pea flowers. This study will provide insight into the composition of anthocyanin in butterfly pea flowers so that the extract can be applied more effectively.

## 2. Results and Discussion

The identification and assignment of anthocyanin peaks was mainly performed based on the comparison between their retention times (RT) and mass spectrometry data with standards, references [28] as shown in Table 1. The chemical structures and molecular weights of six common anthocyanidins and the most common sugars and acylation groups are shown on the basis of preferences. The study of anthocyanin composition in butterfly pea flowers was started by using the basic anthocyanin structure as a reference, taking into account previous literature data on anthocyanin structure [29,30,31,32,33].

**Figure 1:**
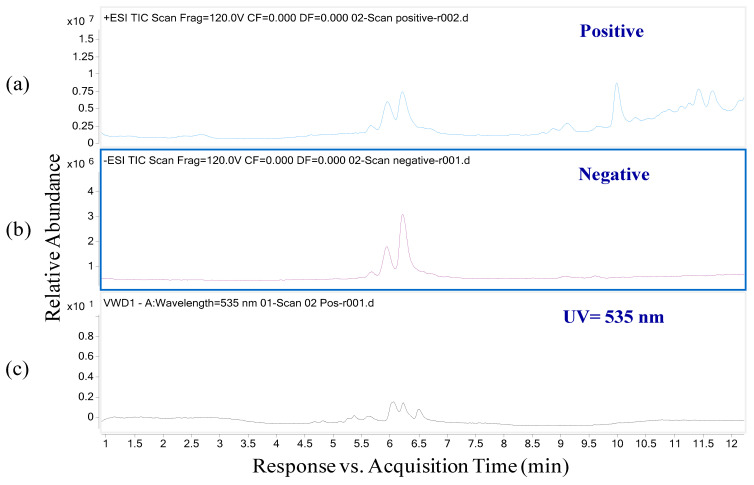
Total ion chromatograms in positive mode (**a**) and negative mode (**b**) and the corresponding chromatogram of UV at 535 nm (**c**).

UPLC chromatograms (Figure 1) for total ions in butterfly pea flowers were collected. It was observed that the acquisition times of anthocyanin peaks ranged from 5.5 to 7.0 min, approximately, in mass chromatogram straight after UV absorption. Unlike other groups of flavonoids or polyphenols, anthocyanins exist in the cationic form, so a positive ion mode has often been used to analyze them. Due to the positive charge and phenolic groups of anthocyanins, these compounds can readily donate protons to free radicals. However, it is difficult to distinguish anthocyanins from flavonol glycosides in active ionization mode using MS, for example between delphinidin glycoside and quercetin glycoside, or between cyanidin glycoside and kaempferol glycoside. These flavonoids may have the same molecular ions and mass fragmentation patterns as the corresponding anthocyanins.

Meanwhile, unlike the MS data collected in the positive ionization mode, the mass spectrometry data obtained with negative ionization provide a wide range of ions that are specific for anthocyanins [34,35]. Therefore, in this work, mass chromatograms of anthocyanins in both positive ion mode and negative ion mode were investigated.

The results in Figure 1a,b also show that in the negative ion mode, the total ion chromatogram for anthocyanins is simpler than in the positive mode. Additionally, the results also show that all ions exhibited the maximum UV absorption at 535 nm, which is consistent with the previous study by Wang et al. [36] on anthocyanins in the black tomato variety Indigo Rose. Therefore, the determination wavelength was 535 nm for all anthocyanins and the UV chromatogram for butterfly pea flowers extract was at 535 nm (Figure 1c).

**Figure 2:**
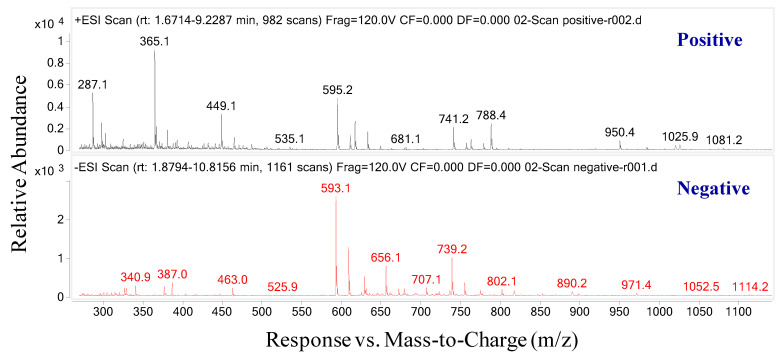
Total ion spectra in positive and negative modes.

To ensure efficiency and accuracy, a combination of spectrophotometric methods, ultraviolet/visible (UV/Vis) and mass spectrometry (MS) methods were performed for anthocyanin identification. Therefore, after UV chromatographic analysis, mass spectrometry data for total ions in both positive and negative modes were also acquired (Figure 2). To obtain the detailed structures of the detected anthocyanins, the MS/MS fragments of anthocyanins were further analyzed. The literature was also used concurrently to focus on and narrow down specific compounds.

**Figure 3:**
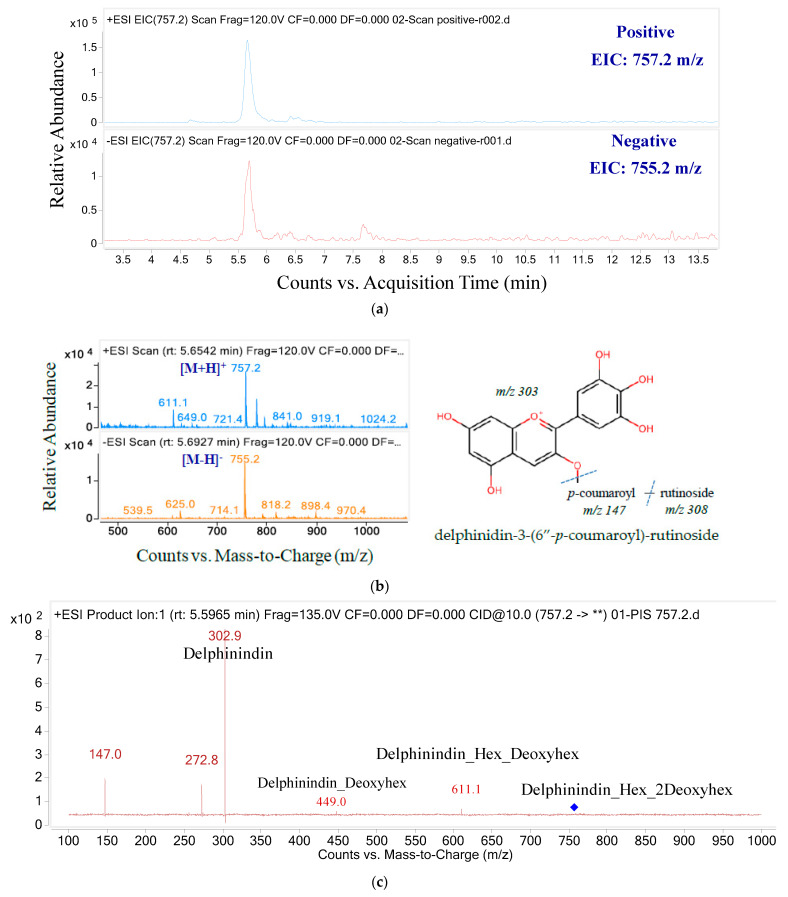
The ion chromatograms (**a**) and ion spectra (**b**) and MS/MS fragments (**) of parent mass 757.2 (**c**).

**Figure 4:**
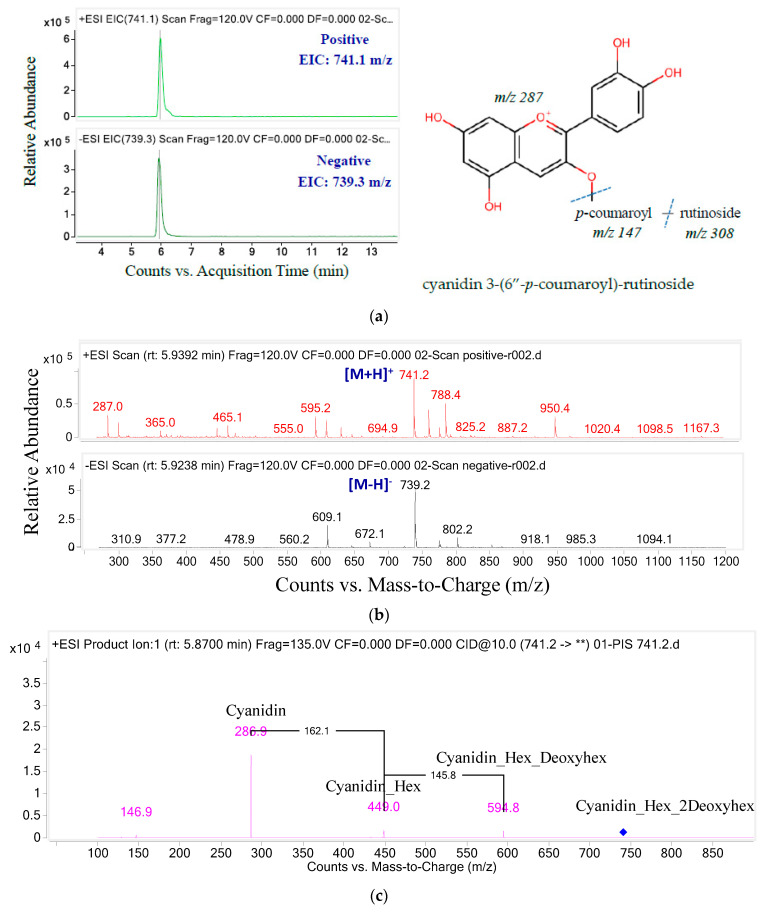
The ion chromatograms (**a**) and ion spectra (**b**) and MS/MS fragments (**) of parent mass 741.2 (**c**).

**Figure 5:**
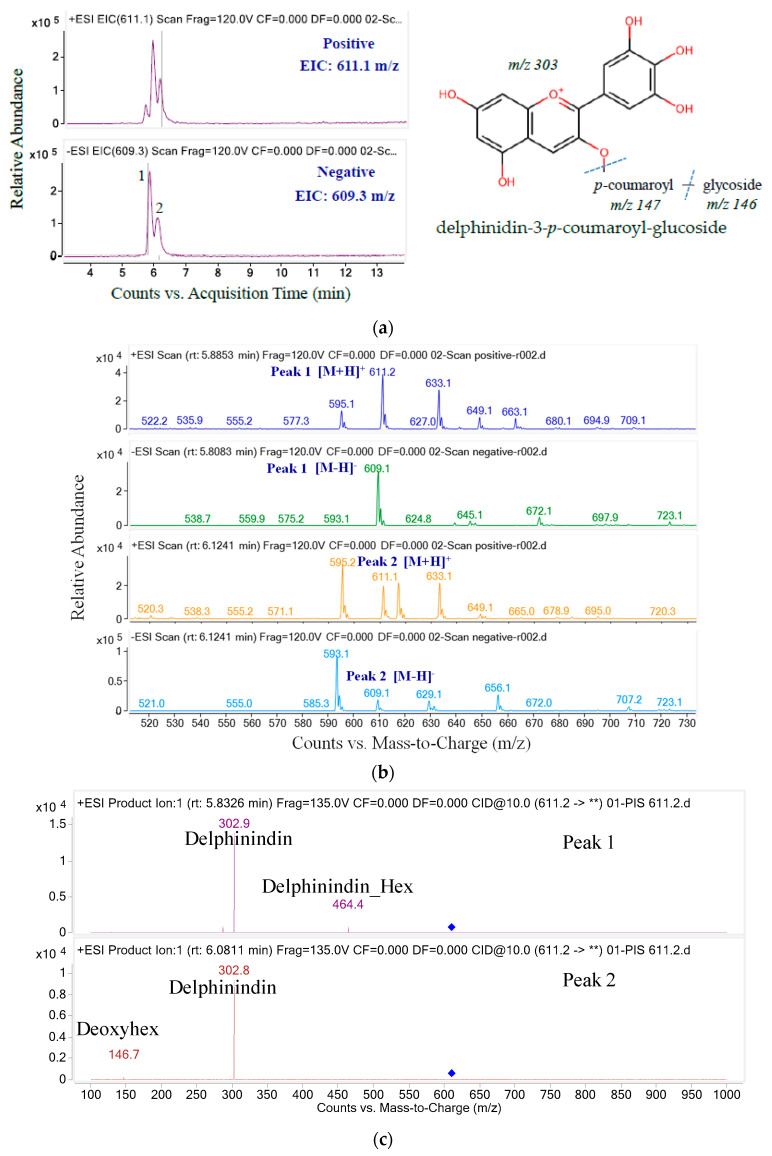
The ion chromatograms (**a**) and ion spectra (**b**) and MS/MS fragments (**) of parent mass 611.2 (**c**).

**Figure 6:**
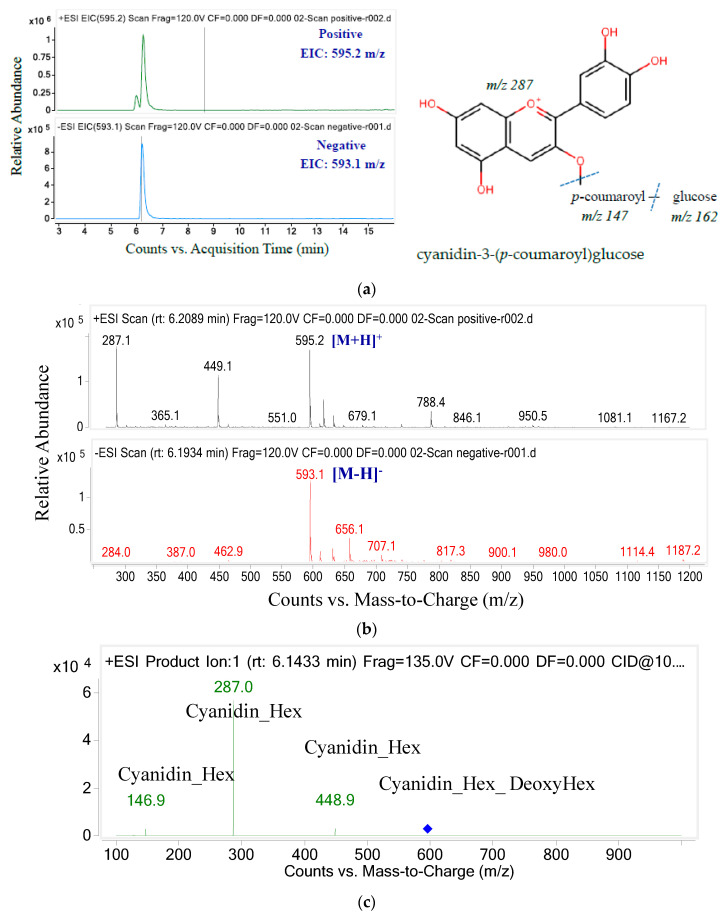
The ion chromatograms (**a**) and ion spectra (**b**) and MS/MS fragments of parent mass 595.2 (**c**).

**Figure 7:**
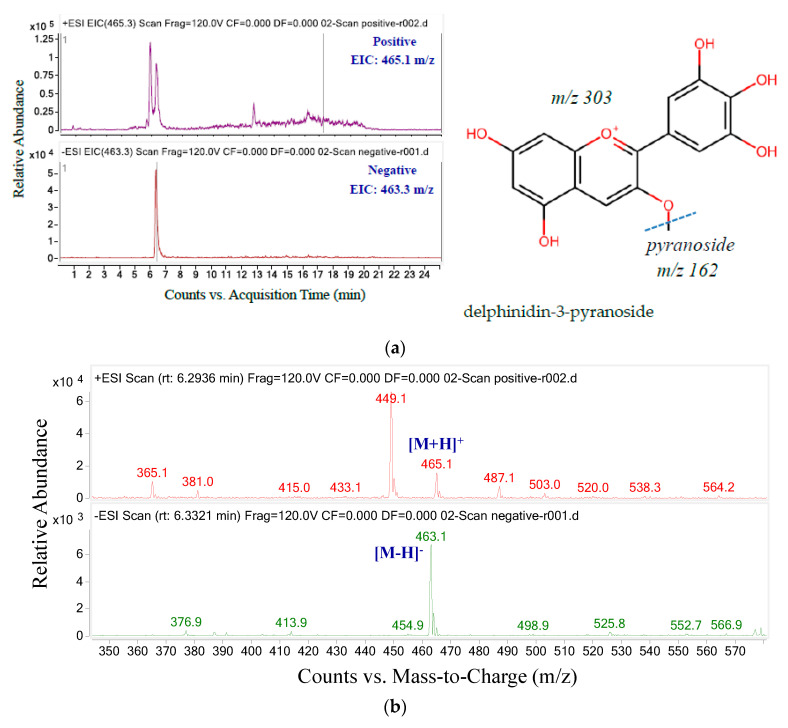

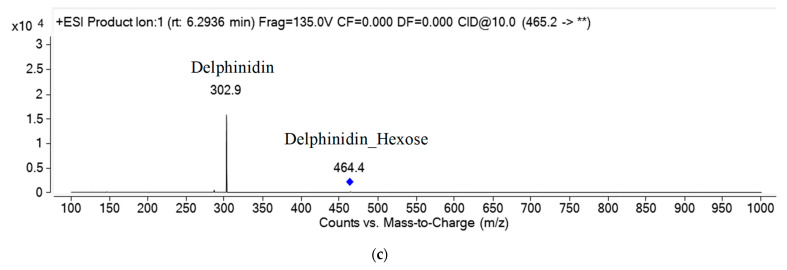
The ion chromatograms (**a**) and ion spectra (**b**) and MS/MS fragments (**) of parent mass 465.2 (**c**).

The structure of anthocyanins was determined by the anthocyanin molecular weight ion [M + H]^+^ and the backbone anthocyanidin molecular weight ion (MS/MS). The *m*/*z* ratios and UPLC-MS/MS ion graphs of each parent ion and their daughter fragments are shown in Figure 3, Figure 4, Figure 5, Figure 6 and Figure 7 and Table 2. Five anthocyanins were identified, including three delphinidin derivatives and two cyanidin derivatives, responsible for the blue color of butterfly pea flowers. The ion chromatograms of the MS/MS product in positive and negative mode and the spectrum of the first anthocyanin eluent component shown in Figure 3 are similar.

Compound 1 at RT 5.62 with [M + H]^+^ *m/z* 757.2 and [M + H]^−^*m*/*z* 755.2 yielded MS2 fragments at *m*/*z* 611.1, 449, 302.9, 272.8 and 147. The fragmentation of *m*/*z* 611.1 can be transiently explained from the parent ion due to the loss of the pentose group, while *m*/*z* 449 can be explained by the loss of 162 Da from the precursor ion *m*/*z* 611, possibly due to the loss of a hexose residue. The rest of the structure (*m*/*z* fragment 147), obtained from the parent ion *m*/*z* 752.7, is shown in Figure 3b.

The *m*/*z* transition from 757.2 to 302.9 and 147 confirms that the aglycone is delphinidin (*m*/*z* 303), and that the compound has p-coumaroyl (*m*/*z* 147). In addition, a fragment with *m*/*z* 611 showed that rutinose (*m*/*z* 308) was linked to delphinidin. This evidence suggests that compound 1 is delphinidin-3-(6″-*p*-coumaroyl)-rutinoside [37].

Showing a similar signal to the positive ion analysis, the negative ion electrospray tandem mass chromatogram and the spectrum of the second anthocyanin are shown in Figure 4. Compound 2 exhibited a molecular ion [M + H]^+^ with *m*/*z* 741.1 and produced a major fragment with *m*/*z* 286.9 that may correspond to cyanidin (*m*/*z* 287). In addition, the *m*/*z* transition from 594.9 to 286.9 implied the loss of rutinose (*m*/*z* 308). Thus, with a p-coumaroyl fragment (*m*/*z* 147), this compound could be tentatively identified as cyanidin 3-(6″-*p*-coumaroyl)-rutinoside [37].

Three peaks can be observed in the chromatogram in the positive ion mode, but only two in the negative ion mode (Figure 5). That means that the third anthocyanin has two isomers. Peaks 1 and 2 have the same daughter fragment of delphinidin aglycone (*m*/*z* 302.9 and 302.8). In peak 1, the *m*/*z* transition from 464.4 to 302.9 indicates a loss of glucose. Meanwhile, peak 2 produces the main fragment, *m*/*z* 146.7, corresponding to *p*-coumaroyl. In addition, because the *cis*-*p*-coumaroyl derivative has a higher polarity, it elutes earlier than its trans configuration [38]. Therefore, peak 1 was tentatively identifiedas delphinidin-3-(*cis-p*-coumaroyl-glucoside) and peak 2 was provisionally identified as delphinidin-3-(*trans-p*-coumaroyl-glucoside) [39].

The chromatograms and MS/MS negative and positive ion electrospray of the final anthocyanin are shown in Figure 6. Two peaks appeared in the mass chromatogram in positive ion mode, but only one peak appeared in negative ion mode. Compound 4 showed molecular ion [M + H]^+^ with *m*/*z* 595.2 and produced two major fragments with *m*/*z* 287.0 (cyanidin) and *m*/*z* 146.9 (*p*-coumaroyl). Together with the *m*/*z* transition from 448.9 to 287.0, indicating glucose loss (*m*/*z* 162), peak 4 was identified as cyanidin-3-(*p*-coumaroyl)-glucose [39].

Similarly, there is a difference between the ion chromatograms in positive and negative ion modes for the final anthocyanins with three and two peaks, respectively (Figure 7). The molecular ion [M + H]^+^ of compound 5 produced a major fragment with *m*/*z* 302.9, corresponding to delphinidin aglycone (*m*/*z* 303). The m/z transition from 464.4 to 302.9 indicated a loss of pyranose (*m*/*z* 162). Therefore, peak 5 was expected to be recognized as delphinidin-3-pyranoside (delphinidin-3-glucoside or delphinidin-3-galactoside) [39].

**Figure 8:**
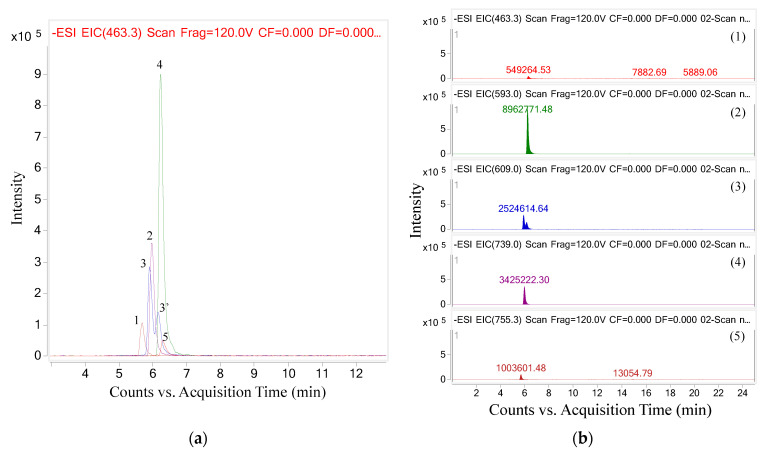
UPLC-MS/MS profile of five anthocyanins found in butterfly pea flower (**a**) and the comparison of their intensity (**b**). (**1**) Delphinidin-3-(6″-p-coumaroyl)-rutinoside, (**2**) Cyanidin 3-(6″-p-coumaroyl)-rutinoside, (**3**) Delphinidin-3-(cis-p-coumaroyl-glucoside), (**3′**) Delphinidin-3-(trans-p-coumaroyl-glucoside), (**4**) Cyanidin-3-(p-coumaroyl)glucose and (**5**) Delphinidin-3-pyranoside.

In summary, after analyzing the chromatograms and spectra of each ion, a total of five anthocyanins were found in the butterfly pea flowers. These were delphinidin-3-(6″-*p*-coumaroyl)-rutinoside, cyanidin 3-(6″-*p*-coumaroyl)-rutinoside, delphinidin-3-(*p*-coumaroyl) glucose in both *cis* and *trans* isomers, cyanidin-3-(*p*-coumaroyl-glucoside) and delphinidin-3-pyranoside. The modifications of these anthocyanins were mainly glycosylation and acylation. In other studies, some delphinidin derivatives were also found in butterfly pea flowers but these compounds had larger sizes, as evidenced by the larger *m*/*z* values of the [M + H]^+^ ion, for example 950, 1296, 1534 [1] or 830.21, 1551.42, 903.22 [22]. In particular, cyanidin derivatives have not been detected/discovered in previous studies. In addition, based on their intensity, we found that cyanidin-3-(*p*-coumaroyl-glucoside) was the most abundant anthocyanin, followed by cyanidin-3-(6″-*p*-coumaroyl)-rutinoside, delphinidin-3-(*p*-coumaroyl-glucoside), delphinidin-3-(6″-*p*-coumaroyl)-rutinoside, and finally delphinidin-3-pyranoside (Figure 8).

## 3. Materials and Methods

### 3.1. Chemicals and Materials

HPLC methanol, acetonitrile, formic acid, and acetic acid were purchased from Merck KGaA, Darmstadt, Germany. All chemicals were analytical grade. Milli-Q water (Milli-Q IQ 7003/7005, Merck, NJ, USA) was used.

Butterfly pea flowers were grown at the College of Agriculture, Can Tho University, Vietnam. Butterfly pea flowers were freeze-dried in an Alpha 2–4 DL freeze dryer (Martin Christ, Germany) at −80 °C and 0.001 mbar to 3−5% moisture content and finely ground before extraction.

### 3.2. Ultra Performance Liquid Chromatography/Ultraviolet/Mass Spectrometry (UPLC/UV/MS) Analysis

#### 3.2.1. Extraction

An amount of 5g lyophilized butterfly pea powder was extracted with 50 mL methanol:water (60:40, *v/v*) using sonication (WUC-A10H, Daihan, Korea) for 60 min at room temperature (23−25 °C). The slurry mixture was centrifuged at 16,000× *g* for 30 min (Z323K, Hermle, Germany).

#### 3.2.2. Purification Procedure

The extraction solution (100 µL) prepared above was put into a centrifuge tube and 700 µL of ice ethanol was added. The tubes were vortexed for 15 s and kept at −80 °C for 60 min. The tubes were then centrifuged at 21,000× *g* for 30 min. The supernatant was filtered through a 17 mm (0.2 mm) PVDF syringe filter (VWR Scientific, Seattle, WA, USA) and dried at 40 °C under vacuum.

The residue was activated with 6 mL of methanol 100%. This mixture was passed through 200 mg C18 solid-phase extraction cartridge (Water, MA, USA) and washed with water (6 mL), then eluted with methanol (6 mL). The methanol fraction containing the parent anthocyanins was concentrated to dryness, and dissolved before HPLC analysis.

#### 3.2.3. The UPLC/UV/MS Conditions

Anthocyanins and its derivatives were determined using LC-ESI-QQQ (6460 Triple Quadrupole System, Agilent, CA, USA) in combination with a UV detector (1260 Infinity, Agilent, CA, USA). Both positive and negative ion electrode mass spectra and tandem mass spectra were recorded. The injection volume was 3.0 µL. Separations of anthocyanins and anthocyanidin aglycones were performed on analytical column Zorbaz Eclipse C18 (2.1 × 50.0 mm, 1.8 μm, Agilent, CA, USA). The mobile phase consisted of water (solvent A) and acetonitrile (solvent B) each containing 0.1% formic acid. The flow rate was 0.3 mL/min and the gradients between the time points were as follows: 0–5 min, 0–30%B; 5–8 min, 30–75%B; 8–25 min, 75–100%B. UV-Visible absorption spectra of anthocyanins were recorded at 535 nm. The MS conditions were as follow: gas temperature, 275 °C; gas flow, 8 L/min; Nebulizer, 45 psi; sheath gas temperature, 350 °C; sheath gas flow: 12 L/min; capillary: 3500 V (positive), 3500V (negative); Nozzle voltage: 500 V (positive), 500V (negative); and scan mass: 270–1000 (*m*/*z*) (positive).

## 4. Conclusions

The mass spectrometry behaviors of anthocyanins in positive and negative ionic modes were studied and were demonstrated to be a valuable tool for identifying anthocyanins from butterfly pea flowers. A new strategy was developed based on observation to distinguish anthocyanin compounds in this flower. Data were generated from UPLC/MS using a developed method that is able to provide rapid and reliable anthocyanin determination with the use of a UV detector. The obtained results show the potential of butterfly pea flowers in anthocyanin extraction and further use as safe colorant in food processing.

## Figures and Tables

**Table 1:** Chemical structures and molecular weight of six common anthocyanidins.

Six Common Anthocyanidins:	Anthocyanidin	R_1_	R_2_	R_3_	MW
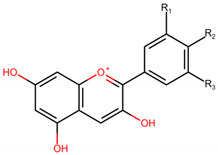	Pelargonidin	H	OH	H	271
	Cyanidin	OH	OH	H	287
	Delphinidin	OH	OH	OH	303
	Peonidin	OMe	OH	H	301
	Petunidin	OMe	OH	OH	317

**Table 2:** Anthocyanin compounds identified in butterfly pea flower.

Peak	Molecular Ion [M + H]^+^ (*m/z*)	Detected Fragments (MS/MS)	Anthocyanins
1	757.2	611.1; 449.0; 302.9; 272.8; 147.0	Delphinidin-3-(6″-*p*-coumaroyl)-rutinoside
2	741.1	594.8; 449.0; 286.9; 146.9	Cyanidin 3-(6″-*p*-coumaroyl)-rutinoside
3	611.2	302.8; 146.7	Delphinidin-3-(*cis*-*p*-coumaroyl-glucoside)
3′	611.1	464.4; 302.9	Delphinidin-3-(*trans*-*p*-coumaroyl-glucoside)
4	595.2	448.9; 287.0; 146.9	Cyanidin-3-(*p*-coumaroyl)glucose
5	465.1	302.9	Delphinidin-3-pyranoside

## Data Availability

Not applicable.

## References

[1] Nair V., Bang W.Y., Schreckinger E., Andarwulan N., Cisneros-Zevallos L. (2015). Protective Role of Ternatin Anthocyanins and Quercetin Glycosides from Butterfly Pea (*ClitoriaTernatea* Leguminosae) Blue Flower Petals against Lipopolysaccharide (LPS)-Induced Inflammation in Macrophage Cells. J. Agric. Food Chem.

[2] Campbell S.M., Pearson B., Marble S.C. (2019). Butterfly Pea (*ClitoriaTernatea*) Flower Extract (BPFE) and Its Use as a PH-Dependent Natural Colorant. EDIS.

[3] Kazuma K., Noda N., Suzuki M. (2003). Flavonoid Composition Related to Petal Color in Different Lines of *Clitoria ternatea*. Phytochemistry.

[4] Rein M. (2005). Copigmentation Reactions and Color Stability of Berry Anthocyanins. Ph.D. Thesis.

[5] Chu B.-S., Wilkin J., House M., Roleska M., Lemos M. (2016). Effect of Sucrose on Thermal and PH Stability of *ClitoriaTernatea* Extract. Int. J. Food Process. Technol.

[6] Siti Azima A.M., Noriham A., Manshoor N. (2017). Phenolics, Antioxidants and Color Properties of Aqueous Pigmented Plant Extracts: ArdisiaColorata Var. Elliptica, *ClitoriaTernatea*, Garcinia Mangostana and SyzygiumCumini. J. Funct. Foods.

[7] Horbowicz M., Kosson R., Grzesiuk A., Dębski H. (2008). Anthocyanins of Fruits and Vegetables—Their Occurrence, Analysis and Role in Human Nutrition. J. Fruit Ornam. Plant Res..

[8] Wu X., Beecher G.R., Holden J.M., Haytowitz D.B., Gebhardt S.E., Prior R.L. (2006). Concentrations of Anthocyanins in Common Foods in the United States and Estimation of Normal Consumption. J. Agric. Food Chem.

[9] Routray W., Orsat V. (2011). Blueberries and Their Anthocyanins: Factors Affecting Biosynthesis and Properties. Compr. Rev. Food Sci. Food Saf..

[10] Barnes J.S., Nguyen H.P., Shen S., Schug K.A. (2009). General Method for Extraction of Blueberry Anthocyanins and Identification Using High Performance Liquid Chromatography–Electrospray Ionization-Ion Trap-Time of Flight-Mass Spectrometry. J. Chromatogr. A.

[11] Smeriglio A., Barreca D., Bellocco E., Trombetta D. (2016). Chemistry, Pharmacology and Health Benefits of Anthocyanins: Anthocyanins and Human Health. Phytother. Res..

[12] Fanali C., Dugo L., D’Orazio G., Lirangi M., Dachà M., Dugo P., Mondello L. (2011). Analysis of Anthocyanins in Commercial Fruit Juices by Using Nano-Liquid Chromatography-Electrospray-Mass Spectrometry and High-Performance Liquid Chromatography with UV-Vis Detector: Liquid Chromatography. J. Sep. Sci..

[13] Castañeda-Ovando A., Pacheco-Hernández M.d.L., Páez-Hernández M.E., Rodríguez J.A., Galán-Vidal C.A. (2009). Chemical Studies of Anthocyanins: A Review. Food Chem..

[14] De Pascual-Teresa S., Moreno D.A., García-Viguera C. (2010). Flavanols and Anthocyanins in Cardiovascular Health: A Review of Current Evidence. Int. J. Mol. Sci..

[15] Clifford M.N. (2000). Anthocyanins—Nature, Occurrence and Dietary Burden. J. Sci. Food Agric..

[16] Lee J., Rennaker C., Wrolstad R.E. (2008). Correlation of Two Anthocyanin Quantification Methods: HPLC and Spectrophotometric Methods. Food Chem..

[17] Khoo H.E., Azlan A., Tang S.T., Lim S.M. (2017). Anthocyanidins and Anthocyanins: Colored Pigments as Food, Pharmaceutical Ingredients, and the Potential Health Benefits. Food Nutr. Res..

[18] Terahara N., Saito N., Honda T., Toki K., Osajima Y. (1989). Structure of Ternatin D1, an Acylated Anthocyanin from Flowers. Tetrahedron Lett..

[19] Terahara N., Saito N., Honda T., Toki K., Osajima Y. (1990). Further Structural Elucidation of the Anthocyanin, Deacylternatin, from *Clitoria ternatea*. Phytochemistry.

[20] Terahara N., Oda M., Matsui T., Osajima Y., Saito N., Toki K., Honda T. (1996). Five New Anthocyanins, Ternatins A3, B4, B3, B2, and D2, from *ClitoriaTernatea* Flowers. J. Nat. Prod..

[21] Terahara N., Toki K., Saito N., Honda T., Matsui T., Osajima Y. (1998). Eight New Anthocyanins, Ternatins C1−C5 and D3 and Preternatins A3 and C4 from Young *ClitoriatErnatea* Flowers. J. Nat. Prod..

[22] Escher G.B., Wen M., Zhang L., Rosso N.D., Granato D. (2020). Phenolic Composition by UHPLC-Q-TOF-MS/MS and Stability of Anthocyanins from *Clitoria ternatea* L. (Butterfly Pea) Blue Petals. Food Chem..

[23] Srivastava A., Akoh C.C., Yi W., Fischer J., Krewer G. (2007). Effect of Storage Conditions on the Biological Activity of Phenolic Compounds of Blueberry Extract Packed in Glass Bottles. J. Agric. Food Chem..

[24] Beaulieu J.C., Stein-Chisholm R.E., Lloyd S.W., Bett-Garber K.L., Grimm C.C., Watson M.A., Lea J.M. (2017). Volatile, Anthocyanidin, Quality and Sensory Changes in Rabbiteye Blueberry from Whole Fruit through Pilot Plant Juice Processing: Blueberry Juice Qualities through Pilot Plant Processing. J. Sci. Food Agric..

[25] Nováková L., Spáčil Z., Seifrtová M., Opletal L., Solich P. (2010). Rapid Qualitative and Quantitative Ultra High Performance Liquid Chromatography Method for Simultaneous Analysis of Twenty Nine Common Phenolic Compounds of Various Structures. Talanta.

[26] Ortega N., Romero M.-P., Macià A., Reguant J., Anglès N., Morelló J.-R., Motilva M.-J. (2010). Comparative Study of UPLC–MS/MS and HPLC–MS/MS to Determine Procyanidins and Alkaloids in Cocoa Samples. J. Food Compos. Anal..

[27] Ceymann M., Arrigoni E., Schärer H., Baumgartner D., Nising A.B., Hurrell R.F. (2011). Rapid High *Perform.* Screening Method Using UHPLC-MS to Quantify 12 Polyphenol Compounds in Fresh Apples. Anal. Methods.

[28] Wu X., Prior R.L. (2005). Identification and Characterization of Anthocyanins by High-Performance Liquid Chromatography−Electrospray Ionization−Tandem Mass Spectrometry in Common Foods in the United States: Vegetables, Nuts, and Grains. J. Agric. Food Chem..

[29] Hrazdina G., Iredale H., Mattick L.R. (1977). Anthocyanin Composition of Brassica Oleracea Cv. Red Danish. Phytochemistry.

[30] Idaka E., Suzuki K., Yamakita H., Ogawa T., Kondo T., Goto T. (1987). Structure of Monoacylated Anthocyanins Isolated from Red Cabbage, *Brassica oleracea*. Chem. Lett..

[31] Fenger J.-A., Sigurdson G.T., Robbins R.J., Collins T.M., Giusti M.M., Dangles O. (2021). Acylated Anthocyanins from Red Cabbage and Purple Sweet Potato Can Bind Metal Ions and Produce Stable Blue Colors. Int. J. Mol. Sci..

[32] Giusti M.M., Ghanadan H., Wrolstad R.E. (1998). Elucidation of the Structure and Conformation of Red Radish (*Raphanus Sativus*) Anthocyanins Using One- and Two-Dimensional Nuclear Magnetic Resonance Techniques. J. Agric. Food Chem..

[33] Mazza G., Mazza G., Miniati E. (2018). Anthocyanins in Fruits, Vegetables, and Grains.

[34] Hua Y., Wainhaus S.B., Yang Y., Shen L., Xiong Y., Xu X., Zhang F., Bolton J.L., Breemen R.B. (2001). Comparison of Negative and Positive Ion Electrospray Tandem Mass Spectrometry for the Liquid Chromatography Tandem Mass Spectrometry Analysis of Oxidized Deoxynucleosides. J. Am. Soc. Mass Spectrom..

[35] Sun J., Lin L., Chen P. (2012). Study of the Mass Spectrometric Behaviors of Anthocyanins in Negative Ionization Mode and Its Applications for Characterization of Anthocyanins and Non-Anthocyanin Polyphenols: Mass Spectrometric Study of Anthocyanins in Negative Ion Mode. Rapid Commun. Mass. Spectrom..

[36] Wang H., Sun S., Zhou Z., Qiu Z., Cui X. (2020). Rapid Analysis of Anthocyanin and Its Structural Modifications in Fresh Tomato Fruit. Food Chem..

[37] Blázovics A., Kursinszki L., Papp N., Kleiner D., Szőke E., Hegyi G., Szilvás A. (2016). Is Professional Prescription of a Commercially Derived Dietary Supplement in Colectomysed Patients Necessary?. Eur. J. Integr. Med..

[38] Wang S., Chu Z., Ren M., Jia R., Zhao C., Fei D., Su H., Fan X., Zhang X., Li Y. (2017). Identification of Anthocyanin Composition and Functional Analysis of an Anthocyanin Activator in Solanum Nigrum Fruits. Molecules.

[39] Stein-Chisholm R., Beaulieu J., Grimm C., Lloyd S. (2017). LC–MS/MS and UPLC–UV Evaluation of Anthocyanins and Anthocyanidins during Rabbiteye Blueberry Juice Processing. Beverages.

